# Genome-Wide Association Analysis of Fusarium Head Blight Resistance in Chinese Elite Wheat Lines

**DOI:** 10.3389/fpls.2020.00206

**Published:** 2020-02-27

**Authors:** Zhanwang Zhu, Ling Chen, Wei Zhang, Lijun Yang, Weiwei Zhu, Junhui Li, Yike Liu, Hanwen Tong, Luping Fu, Jindong Liu, Awais Rasheed, Xianchun Xia, Zhonghu He, Yuanfeng Hao, Chunbao Gao

**Affiliations:** ^1^Hubei Key Laboratory of Food Crop Germplasm and Genetic Improvement, Food Crops Institute, Hubei Academy of Agricultural Sciences/Hubei Engineering and Technology Research Center of Wheat/Wheat Disease Biology Research Station for Central China, Wuhan, China; ^2^Institute of Crop Sciences, Chinese Academy of Agricultural Sciences (CAAS), Beijing, China; ^3^Department of Plant Sciences, North Dakota State University, Fargo, ND, United States; ^4^Institute of Plant Protection and Soil Science, Hubei Academy of Agricultural Sciences, Wuhan, China; ^5^CIMMYT-China Office, Beijing, China; ^6^Hubei Collaborative Innovation Center for Grain Industry, Yangtze University, Jingzhou, China

**Keywords:** *Fusarium graminearum*, genome-wide association study, marker-assisted selection, single nucleotide polymorphism, *Triticum aestivum*

## Abstract

Fusarium head blight (FHB) is a devastating wheat disease worldwide. To decipher the genetic architecture of FHB resistance in Chinese germplasm, a Wheat Association Panel for Scab Research (WAPS) consisting of 240 leading Chinese wheat cultivars and elite lines was genotyped using the 90K single nucleotide polymorphism (SNP) arrays. The FHB response was evaluated in the field nurseries in Wuhan in Hubei Province over four consecutive years from 2014 to 2017. Five quantitative trait loci (QTL) were consistently identified on chromosome arms 1AS, 2DL, 5AS, 5AL, and 7DS using a mixed linear model (MLM), explaining 5.6, 10.3, 5.7, 5.4, and 5.6% of phenotypic variation, respectively. The QTL on 5AS, 5AL, and 7DS QTL are probably novel. The allelic frequency analysis indicated that cultivars from the Middle and Lower Yangtze River Valleys harbored more favorable alleles and were therefore more resistant than those from other regions. To facilitate in-house germplasm screening and marker-assisted selection (MAS), SNP-derived PCR markers were developed for the QTL regions on 1AS, 5AS, and 5AL QTL. In addition to the above five QTL, the WAPS population had a very low frequency of *Fhb1*, confirming that the gene is not widely used in Chinese wheat breeding programs. The resistant lines and molecular markers developed in this study are resources and information for enhancing FHB resistance in breeding populations by marker-assisted recurrent selection and gene stacking.

## Introduction

Fusarium head blight, caused mainly by the fungus *Fusarium graminearum* Schwabe, is a devastating disease of wheat worldwide, particularly in warm and humid regions (Bai et al., 2018). FHB greatly reduces grain yield and end-use quality (Bai and Shaner, 1994; Dexter et al., 1996, 1997). Infected grains contain *Fusarium* toxins, mainly B type trichothecenes, such as DON, 3-O-acetyl-DON (3-AcDON), 15-O-acetyl-DON (15-AcDON), DON-3-G, and NIV, making grain unsuitable for food and feed (McMullen et al., 1997; Foroud et al., 2019; Xu et al., 2019). The incidence and severity of FHB in China, particularly in the Yellow and Huai River Valley, have increased over the last two decades (Ma et al., 2016), mainly due to the long-time maize-wheat rotation practice, straw retention, reduced tillage, and climate change. Although FHB can be partially managed with agronomic and agrochemical measures, genetic improvement of host resistance remains the best approach for controlling this disease.

Resistance to FHB is a typical quantitative trait and the infection process is complicated. Plant morphological and phenological traits and their growing environments all affect FHB infection (Bai et al., 2018). Buerstmayr et al. (2019) summarized the influence of plant height, AE and heading date/flowering time on FHB response. Taller plant height and higher AE contribute to FHB resistance. Different heading date/flowering time results in differences in weather conditions during disease infection and development, but the differences vary depending on specific environments and could lead to either positive, negative or no correlation to FHB responses. Due to the close correlation between morphological traits and FHB resistance, it is critical to include these traits in FHB resistance studies.

Using linkage analysis, QTL for FHB resistance have been mapped on all 21 wheat chromosomes partitioned into 44 chromosomal regions (Buerstmayr et al., 2009; Liu et al., 2009; Ma et al., 2020). To date, seven FHB genes (*Fhb1–Fhb7*) have been formally cataloged. *Fhb2* on the short arm of 6B, *Fhb4* on the long arm of 4B, and *Fhb5* near the centromere of 5A were finely mapped (Jia et al., 2018). *Qfhs.ifa-5A* from a Sumai 3 derivative CM-82036 was finely mapped as two closely linked QTL *Qfhs.ifa-5Ac* and *Qfhs.ifa-5AS* (Steiner et al., 2019). *Qfhs.ifa-5Ac* overlapped with *Fhb5* according to their mapping intervals.

*Fhb1* on chromosome arm 3BS is the most important and widely studied (Buerstmayr et al., 2009). It was first identified as a *PFT* gene (Rawat et al., 2016) and later as a *TaHRC* or *His* gene located adjacent to *PFT* (Su et al., 2019; Li G. et al., 2019). However, the molecular nature of this gene needs further study (Lagudah and Krattinger, 2019). Functional markers developed from *TaHRC* in the *Fhb1* region were validated as diagnostic and, therefore, useful for MAS of *Fhb1* (Su et al., 2018; Zhu et al., 2018). *QFhb.mgb-2A* originally mapped in a Sumai 3 derived wheat line 02-5B-318 (Giancaspro et al., 2016) was isolated as a *WAK2* gene. The function of *WAK2* for FHB resistance was validated through gene expression comparison between resistant and susceptible wheat lines, and resistance evaluation in three TILLING mutants for WAK protein function (Gadaleta et al., 2019). Another important locus, located on 2DL, was repeatedly identified from different sources of Chinese germplasm, including Wuhan 1 (Somers et al., 2003), CJ9306 (Jiang et al., 2007a, b), Wangshuibai, and CIMMYT line SYN1 (Zhu et al., 2016). This QTL is thought to be transcription factor *TaWRKY70* that mediates expression of downstream metabolite biosynthetic genes *TaACT*, *TaDGK*, and *TaGLI* to condition resistance to *Fusarium graminearum* (Kage et al., 2017a, b). Hu et al. (2019) identified an expression QTL for gene *Traes_2DL_179570792* overlapped with the 2DL QTL interval.

Genome-wide association studies (GWAS) have been used to dissect the genetic basis of complex economic traits in wheat, such as pre-harvest sprouting resistance (Lin et al., 2016; Zhou et al., 2017) and grain yield (Sun et al., 2017; Liu J. et al., 2017; Sukumaran et al., 2015). However, very few GWAS on wheat have addressed FHB resistance. Kollers et al. (2013) conducted a GWAS on FHB response in European winter wheat using 732 microsatellite markers and found significant associations involving all wheat chromosomes except 6B. Arruda et al. (2016) found 10 SNP-trait associations on chromosomes 4A, 6A, 7A, 1D, 4D, and 7D and multiple SNPs associated with *Fhb1* in US winter wheat, including severity, incidence, FHB index, and DON content. Wang et al. (2017) identified QTL on chromosomes 1B, 2B, 4B, 5A, 5B, and 6A in spring wheat lines developed in the Pacific Northwest and at CIMMYT. However, the genetic basis underlying FHB resistance in elite Chinese wheat has not been examined.

In the current study, we analyzed a panel of Chinese wheat cultivars and elite breeding lines including more than 50 widely grown cultivars using the wheat 90K arrays to: (1) study the phenotypic variance of FHB response in Chinese wheat germplasm, and (2) determine the genetic architecture of FHB resistance in the panel. The results should provide a better understanding of the genetic basis and diversity in FHB response of the Chinese cultivars, and facilitate the improvement of FHB resistance levels by stacking FHB resistance QTL using MAS.

## Materials and Methods

### Plant Materials

The WAPS comprised 240 geographically diverse bread wheat (*Triticum aestivum*) cultivars and elite lines from China (Supplementary Table S1). Out of these, 229 entries were obtained from 12 provinces located in five agro-ecological zones (Zhuang, 2003; Li H. et al., 2019), including the Northern Winter Wheat Zone (Zone I), Yellow and Huai River Valleys Facultative Wheat Zone (Zone II), Middle and Lower Yangtze River Valleys Autumn-Sown Spring Wheat Zone (Zone III), Southwestern Autumn-Sown Spring Wheat Zone (Zone IV), and Northeastern Spring Wheat Zone (Zone VIII). The remaining 11 genotypes were introduced from CIMMYT, including seven lines from the 14th FHB Screening Nursery and an Australian cultivar Gamenya. More than 50 cultivars of this panel achieved peak annual planting areas of 1 × 10^5^ hectares from 2000 to 2016, including the founder parents Jimai 22, Ningmai 9, Xiaoyan 6, and Zhoumai 18. Sumai 3 was used as an FHB resistant check, CJ9306 and Wuhan 1 as moderately resistant checks, and Gamenya as a susceptible check.

### Inoculum Preparation

The inoculum comprised two highly aggressive isolates of *F. graminearum*, Huanggang 1 and Fg 5035, collected in Hubei Province. The isolate Huanggang 1 was kindly provided by the Institute of Plant Protection and Soil Science, Hubei Academy of Agricultural Sciences, and Fg 5035 was provided by Huazhong Agricultural University.

The *F. graminearum* was preserved on PDA (potato dextrose agar) medium at −20°C until use. The mycelium was inoculated into a 5% mung bean broth medium to grow *F. graminearum* conidia with shaking at 180 rpm under 28°C for 5–7 days. The flasks content was filtered and the concentration of conidia was examined with blood counting chamber under a microscope.

### Phenotypic Assessment

A combination of type I and type II resistance was assessed in the field nurseries at Nanhu Experimental Station (altitude 27 m, latitude 30.48°N), Food Crops Institute, Hubei Academy of Agricultural Sciences, during the 2013–2014, 2014–2015, 2015–2016, and 2016–2017 cropping seasons, hereafter referred to as 2014, 2015, 2016, and 2017, respectively. The average annual rainfall is about 1,270 mm at this location and the growing season extends from early November to May. The trials were carried out in randomized complete blocks with two replications. Each plot comprised 2 × 1 m rows with 25 cm space between rows.

In 2014, 2015, and 2016, an overhead misting system was applied to increase the humidity to favor pathogen infection and FHB development. The system included 1.5 m high micro-sprinklers spaced at distances of 1.5 × 1.5 m and operated automatically by a programmable timer from 9 am to 7 pm with 2 min of spraying per hour. Ten randomly selected flowering spikes per plot were labeled with blue tapes. About 30 mL of water suspension of *F. graminearum* conidia at a concentration of 50,000 spores/mL were sprayed on the labeled spikes, which were then assessed 21 days post-inoculation for FHB response by counting the number of diseased spikelets and total spikelets per spike. The FHB index (%) was calculated as (Severity × Incidence)/100 (Stack and McMullen, 1994), where incidence is the percentage of FHB infected spikes, and severity is the averaged percentage of symptomatic spikelets, e.g. a line with nine out of ten spikes infected (90% incidence), and 20% of symptomatic spikelets on average (20% severity), had an FHB index of 18% (90% × 20%) (He et al., 2013).

In 2017, the population was planted in a rice field with the same experimental design. Grain-spawn inoculation was conducted by scattering 600 g scabby wheat grains per 100 m^2^ on the soil surface about 1 month before anthesis, and FHB was visually scored 28 days post-anthesis.

The plant heights for all the accessions used in this study were recorded about 2 weeks after anthesis. For each plot, the plant heights were measured two times to calculate the average.

### Genotyping and Quality Control

Genomic DNA for SNP assays was extracted from young leaf tissue for each accession using a modified CTAB procedure. The WAPS population was genotyped using the Illumina wheat 90K SNP arrays (Wang et al., 2014) at the USDA-ARS Small Grains Genotyping Laboratory in Fargo, ND, United States. SNPs were called using Illumina GenomeStudio Software. The accuracy of SNP clustering was validated manually. SNPs with less than 20% missing data and a MAF exceeding 5% were selected for subsequent analysis. Physical positions of the SNP markers were obtained from the International Wheat Genome Sequencing Consortium (IWGSC^1^) database. The marker density was drawn using the CMplot package in R^2^. The flanking markers of 11 known FHB QTL (He et al., 2013) were used to genotype the WAPS population. Markers for 10 of them, *Fhb1* (UMN 10), *Fhb2* (*Xwmc179*), *Qfhs.ifa-5A* (*Xbarc186* and *Xbarc180*), QTL on 3A from Frontana (*Xdupw227*), QTL on 5A from Frontana (*Xbarc197* and *Xgwm129*), QTL on 2DL from Wuhan 1 (*Xwmc245*), QTL on 4BS from Wuhan 1 (*Xwmc238* and *Xgwm149)*, *QFhs.nau-2DL* (*Xgwm157* and *Xgwm539*), *Qfhs.ndsu-3AS* (*Xgwm2*), and *Qfhs.fcu-7AL* (*Xbarc121* and *Xwmc488*) were successfully applied. Diagnostic markers *PFT-CAPS* for *Fhb1* candidate gene *PFT* and *His-InDel* for *Fhb1* casual gene *His* (Zhu et al., 2018) were used to detect *Fhb1* in this panel.

### Statistical Analysis, Population Structure, and GWAS

Comparisons of the means of different groups were done using *t*-tests. For analysis of the FHB index, flowering time, and plant height, best linear unbiased estimates (BLUE) were calculated across the environments using the ANOVA function in IciMapping V4.0 software (Li et al., 2007).

Population structure analysis was performed using ADMIXTURE^3^. The Genomic Association and Prediction Integrated Tool (GAPIT) was used to generate distance-based clustering distribution and scree plots^4^.

Associations between genotypic and phenotypic data were analyzed using Tassel v5.0^5^. The kinship (K)+PCA model was chosen to perform MLM analysis to control background variation and eliminate spurious MTAs. Markers with adjusted –log_10_ (*P*-value) ≥ 3.0 were considered significant markers for FHB resistance (Liu Y. et al., 2017). The *P*-value distributions of markers (observed *P*-values plotted against expected *P*-values) were shown in Q-Q plots. Manhattan and Q-Q plots were drawn using CMplot. To compare the physical positions of the QTL identified in the current study and the known genes/QTL, relevant markers and gene sequences were used to blast Chinese Spring reference genome sequences (IWGSC RefSeq v1.0^6^).

### Development of PCR-Based Markers for Significant SNPs

The SNPs significantly associated with FHB resistance were converted to KASP, CAPS or conventional PCR-based markers to facilitate their application in MAS. Primers for KASP markers were designed by PolyMarker^7^ and fluorescence signals were detected using the multifunctional microplate reader PHERAstar*^*Plus*^* (BMG LABTECH, Ortenberg, Germany) and analyzed using KlusterCaller software (LGC Genomics, Teddington, United Kingdom). The primers for other types of markers were designed based on chromosome-specific polymorphisms among homologous sequences in the target regions obtained from the EnsemblPlants database^8^. The PCR products were visually detected by 1.5% agarose gel electrophoresis.

## Results

### Phenotypic Variation

Analysis of variance of FHB indices showed significant variation for years, genotypes, and genotype-by-year interaction (Table 1). Heritability of FHB indices across the 4 years was 0.63, indicating that the FHB response of the WAPS population was mainly controlled by genetic factors.

**TABLE 1:** Analysis of variance of Fusarium head blight indices in the WAPS (Wheat Association Panel for Scab Research) population.

Source of variation	*Df*	Mean square	*F*-value	*P-*value
Genotype	239	2295.1	11.3	<0.0001
Year	3	186007.8	914.1	<0.0001
Genotype × Year	717	598.0	2.9	<0.0001
Replication/Year	4	5610.1	27.6	<0.0001

**h*_*b*_^2^ = 0.63.*

Disease symptoms were moderate to high in the first 3 years under spray inoculation. The mean FHB indices of the WAPS population were 58.3, 49.2, and 69.3% in 2014, 2015, and 2016, respectively. The highest FHB index was recorded in 2016, probably due to warmer weather during inoculation and disease development. FHB symptom in 2017 was lighter than those in the earlier years, with a mean FHB index of 29.1%. This might be attributed to the grain-spawn inoculation method providing less pathogen pressure than spray inoculation. The variation in symptom levels might cause differences in the frequency distribution of the FHB index. The frequency distribution in the years 2016 and 2017 did not fit a normal curve (Supplementary Figure S1).

The correlation coefficients of FHB indices among the first 3 years ranged from 0.41 to 0.66, while those between 2017 and the earlier years were relatively lower with a range of 0.31–0.52 (Supplementary Table S2).

The BLUE values across 4 years for FHB indices exhibited wide variation ranging from 5.3 to 88.6% (Supplementary Figure S1). Sumai 3 had the best resistance with a BLUE value of FHB indices of 5.3%. The two moderately resistant checks, CJ9306 and Wuhan 1, were rated 21.3 and 27.4%, respectively, whereas the susceptible check Gamenya was 73.8% for the BLUE values of FHB indices across years. Genotypes from Jiangsu and Hubei had an average FHB index of 28.6%; followed by cultivars from Anhui and Gansu, with an index of 39.5 and 39.7%, respectively. Genotypes from Beijing (45.6%), Shaanxi (47.7%), and Sichuan (49.4%) had relatively high values, and those from Shanxi (50.4%), Shangdong (52.7%), Henan (57.3%), Ningxia (61.5%), and Hebei (62.5%) tended to be highly susceptible (Supplementary Figure S2). CIMMYT lines showed moderate resistance with an average FHB index of 39.5%.

### Marker Distribution and Population Structure

Of 81,587 SNPs called in the assay, 22,922 were polymorphic in the population and selected for subsequent analysis. After removing SNPs with MAF <5 and >20% missing data, 19,803 were employed for GWAS. These SNP loci spanned a physical distance of 14,036.0 Mb, with an average density of 0.963 Mb per locus. SNP density for the D genome (1.781 Mb per SNP) was lower than those for the A (0.852 Mb per SNP) and B (0.785 Mb per SNP) genomes (Supplementary Table S3 and Supplementary Figure S3).

**FIGURE 1:**
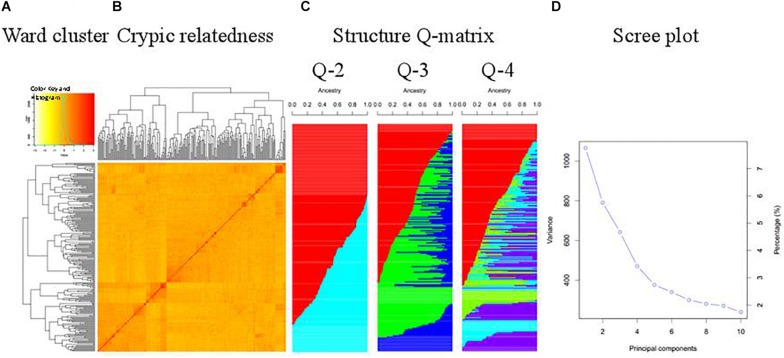
Structure analysis of the WAPS population. **(A)** Ward clustering of the population; **(B)** Cryptic relatedness matrix based on genetic distance; **(C)** Matrices of membership coefficients corresponding to 2 to 4 hypothetical subpopulations; **(D)** Scree plot generated by GAPIT demonstrating genetic variance explained by each principal component.

The WAPS population was divided into three subgroups, essentially consistent with geographic origin and pedigree (Figure 1). For example, Ningmai 16 and Zhenmai 6, both derived from Ningmai 9, clustered with Ningmai 9 in the same subgroup. Most of the wheat cultivars from Zone I, Zone II, Zone VIII, and CIMMYT were placed in subgroup I (marked in red in Q-3 in Figure 1C), most of the Zone IV cultivars in subgroup II (marked in green), and the majority of Zone III cultivars in subgroup III (marked in blue). There were exceptions – a significant portion of the genotypes from Zone II were allocated to subgroups II and III rather than subgroup I, indicative of germplasm exchange or the artificial nature of zoning. Weak kinship existed among cultivars in the WAPS population – the scree plot demonstrated that the first four PCs explained about 23% of the genetic variation in the panel (Figure 1D).

### Maker-Trait Association Analysis and Geographical Distribution of Favorable Alleles

**FIGURE 2:**
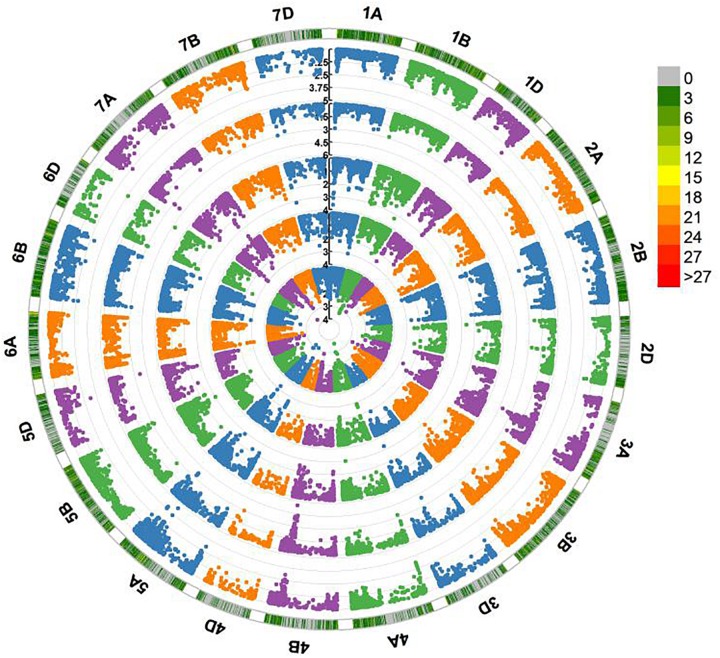
Circle Manhattan plots revealing significant QTL for Fusarium head blight resistance in 240 wheat accessions performed by Tassel v5.0 using the MLM model. Traits plotted from inside to outside are FHB indices in 2014, 2015, 2016, 2017, and the BLUE value across 4 years. The –log_10_ (*P*) values from a genome-wide scan are plotted against positions on each of the 21 chromosomes. Marker density along each chromosome is shown with different colors in the outermost circle. Numbers in the legend represent the number of SNPs within a 1 Mb window size.

Five QTL on chromosome arms 1AS, 2DL, 5AS, 5AL, and 7DS, designated *QFhb.hbaas-1AS*, *QFhb.hbaas-2DL*, *QFhb.hbaas-5AS*, *QFhb.hbaas-5AL*, and *QFhb.hbaas-7DS*, respectively, were significant in at least two environments, explaining phenotypic variation ranging from 4.9 to 10.3% (Table 2, Figure 2 and Supplementary Figure S4). Representative significant markers were *IWB75039*, *Xgwm539*, *IWB21456*, *IWB42293*, and *IWB15569*, respectively.

**TABLE 2:** Loci significantly associated with FHB resistance in at least two environments identified in 240 wheat accessions using the MLM model in Tassel v5.0.

Marker^a^	Variant^b^	Chr^c^	Physical position^d^ (Mb)	Environment	*P-*value	*R*^2e^ (%)	*P*-value for plant height^f^	*P*-value for days to flowering^f^
*IWB75039*	A/G	1AS	10.0	2016, 2017	3.12 E-04	5.6, 5.6	0.650	0.113
*Xgwm539*	Wuhan 1 allele/ others	2DL	513.1	2016, BLUE	2.80 E-05	10.3, 7.5	0.258	0.062
*IWB21456*	A/G	5AS	9.6	2014, 2015, BLUE	2.58 E-04	5.0, 5.0, 5.7	0.003	0.608
*IWB42293*	A/G	5AL	540.6	2015, BLUE	4.68 E-04	4.9, 5.4	0.031	0.110
*IWB15569*	C/T	7DS	23.5	2016, 2017	4.34 E-04	4.9, 5.6	0.367	0.054

*^*a*^Representative markers showing the strongest association with the resistance locus; ^*b*^Favorable allele is underlined; ^*c*^Chr: Chromosome; ^*d*^Physical positions based on the Chinese Spring reference genome sequences from the International Wheat Genome Sequencing Consortium (IWGSC, http://www.wheatgenome.org); ^*e*^Percentage of phenotypic variance explained; ^*f*^*P*-values for association between the representative markers of FHB QTL and plant height and flowering time in WAPS population calculated by Tassel v5.0 using the MLM model.*

**FIGURE 3:**
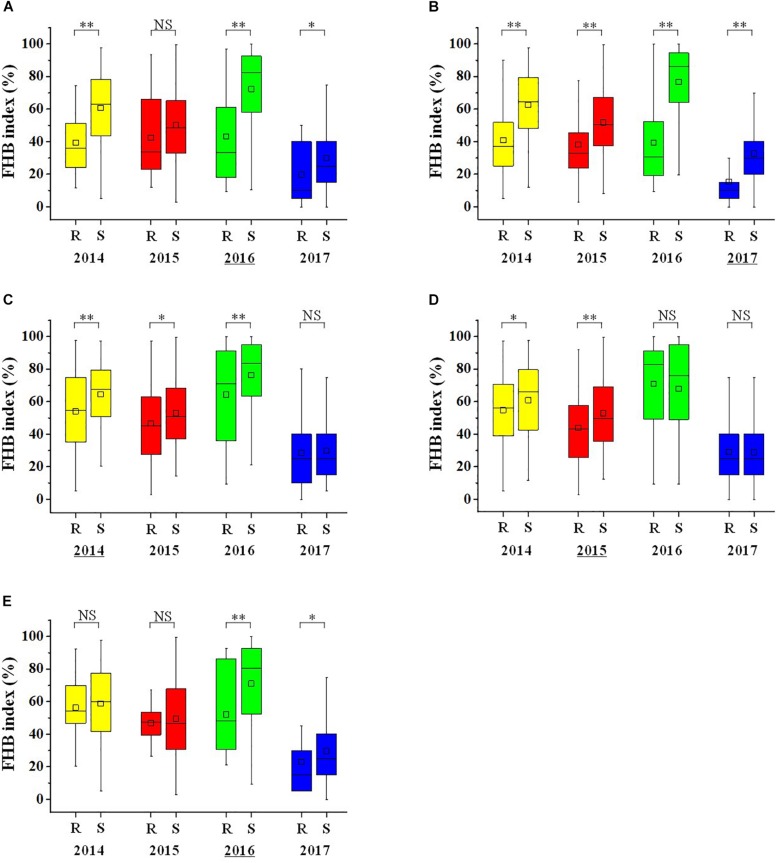
Box plots visualizing differences in Fusarium head blight index between two groups of wheat accessions with contrasting resistance or susceptibility alleles for QTL on chromosomes 1AS **(A)**, 2DL **(B)**, 5AS **(C)**, 5AL **(D)**, and 7DS **(E)**. The yellow, red, green and blue box plots represent 2014, 2015, 2016 and 2017, respectively. The horizontal line within boxes represent the median, and black squares indicate means. ‘^∗∗^’, ‘^∗^’ and “NS,” *P* < 0.01, *P* < 0.05 and non-significant. “R” and “S” represent lines with the resistance and susceptibility alleles, respectively. The year with the highest FHB index difference between R and S for each QTL was underlined in the X-axis.

The mean FHB index of the lines with the favorable allele of *QFhb.hbaas-1AS* was 15.2–40.2% lower than those with the unfavorable allele over the 4 years. Differences were even higher for *QFhb.hbaas-2DL* (26.1–53.1%) (Figure 3). The other three QTL showed smaller effects. *QFhb.hbaas-5AS* and *QFhb.hbaas-7DS* reduced the FHB index by 4.3–16.2% and 4.1–26.6%, respectively, and *QFhb.hbaas-5AL* reduced the FHB index by 10.1% in 2014 and 17.1% in 2015 (Figure 3).

Of the 240 genotypes, 23, 48, 141, 98, and 18 possessed resistance alleles *QFhb.hbaas-1AS*, *QFhb.hbaas-2DL*, *QFhb.hbaas-5AS*, *QFhb.hbaas-5AL*, and *QFhb.hbaas-7DS*, respectively, based on marker analysis (Supplementary Figure S5). More than half of the lines carrying the *QFhb.hbaas-1AS* resistance allele were from Jiangsu and Hubei (Supplementary Figure S5a). The frequency was even higher for *QFhb.hbaas-2DL*: 36 carriers originated from Jiangsu or Hubei (Supplementary Figure S5b). Resistance alleles of the remaining three QTL *QFhb.hbaas-5AS*, *QFhb.hbaas-5AL*, and *QFhb.hbaas-7DS* presented at relatively high frequencies in the cultivars from Henan, Shandong, and Shaanxi in the Yellow and Huai River Valley region (Supplementary Figures S5c–e).

Six cultivars – Sumai 3, Ning 7840, Ningmai 9, Ningmai 13, Ningmai 16, and Zhenmai 5 – carried *Fhb1* based on functional marker analysis of *His-Indel* (Supplementary Figure S6a). These cultivars were developed in Jiangsu (Middle and Lower Yangtze River Valleys, Zone III) where FHB has traditionally been more severe than other regions in China.

The *P-*value for association of a representative marker for *QFhb.hbaas-5AS* with plant height was 0.003 when calculated using the MLM model by Tassel software. This indicated that the FHB QTL was pleiotropic or closely linked with a gene for plant height (Table 2). The other four QTL showed no significant relationship with plant height. All five FHB QTL had no significant effect on flowering time.

### PCR-Based Markers for *QFhb.hbaas-1AS*, *QFhb.hbaas-5AS*, and *QFhb.hbaas-5AL*

The SNPs (*IWB75039*, *IWB21456*, and *IWB42293*) associated with *QFhb.hbaas-1AS*, *QFhb.hbaas-5AS*, and *QFhb.hbaas-5AL* were converted to PCR-based markers and designated *FHB-1AS-PCR*, *FHB-5AS-KASP*, and *FHB-5AL-CAPS*, respectively. *FHB-1AS-PCR* produced no PCR products for the resistance allele, and a 389 bp fragment for the susceptibility allele (Supplementary Figure S6b). For *FHB-5AS-KASP*, red and blue dots represented the resistance and susceptibility alleles of *QFhb.hbaas-5AS*, respectively (Supplementary Figure S6c). The CAPS marker *FHB-5AL-CAPS* produced a 334 bp fragment for both resistance and susceptibility alleles. After digestion with restriction endonuclease *Age*I (recognition sequence: 5′-A/CCGGT-3′), the products of cleaved fragments of 213 and 121 bp were obtained for the susceptibility allele, whereas the resistance allele was represented by the intact 334 bp fragment (Supplementary Figure S6d).

Primers for the three converted markers are listed in Table 3, and protocols for their use are described in Supplementary Table S4. For validation, the three markers were used to genotype 240 WAPS accessions with very low frequencies of inconsistency (3.3, 3.3 and 4.2% for each marker).

**TABLE 3:** Primers for markers *FHB-1AS-PCR*, *FHB-5AS-KASP*, and *FHB-5AL-CAPS*.

Marker	Primer	Sequence (5′-3′)
*FHB-1AS-PCR*	P75039F	GTTTTCAGGGCCTTATCAACTGAAG
	P75039R	GGTTCGGTTGGTGCTTAATCACT
*FHB-5AS-KASP*^a^	P21456A	GAAGGTGACCAAGTTCATGCTATCGACAATTACATCAAATGACTGA
	P21456B	GAAGGTCGGAGTCAACGGATTATCGACAATTACATCAAATGACTGG
	P21456C	AATAACGTGGCTATCAGTGGT
*FHB-5AL-CAPS*	P42293F	GCCAGAGCACTGGTAATTACAGT
	P42293R	CGATTCCGACCACCACGAG

*^*a*^Tails for competitive primers are underlined.*

### Prediction of Candidate Genes

The annotations of the high confidential genes (IWGSC RefSeq annotation v1.0 see text footnote 1) less than 2 Mb away from the representative SNPs of *QFhb.hbaas-1AS*, *QFhb.hbaas-5AS*, *QFhb.hbaas-5AL*, and *QFhb.hbaas-7DS* were examined to determine their candidacy for FHB resistance. Bioinformatics analysis identified four genes encoding 12-oxophytodienoate reductase-like protein, six genes encoding RLK, and two genes corresponding to UGT near *QFhb.hbaas-1AS* (Supplementary Table S5). Two genes encoding pathogenesis-related (PR) protein 1 were considered as candidates for *QFhb.hbaas-5AS*. For *QFhb.hbaas-5AL*, two genes encoding glucan endo-1, 3-beta-glucosidase, one for defensin and one for RLK were identified. A gene encoding enhanced disease resistance 2-like protein might contribute to FHB resistance for *QFhb.hbaas-7DS*.

## Discussion

### FHB Resistance Variation

The WAPS population with the wheat accessions collected from different agro-ecological regions represents a large proportion of the genetic diversity in modern Chinese wheat cultivars. Thus, we can use mean FHB indices in the different regions to assess relative levels of FHB resistance across the regions. Cultivars from Jiangsu and Hubei, in the Middle and Lower Yangtze River Valleys, had the highest levels of FHB resistance, whereas cultivars from other provinces tended to be more susceptible, particularly those from Shandong, Henan, and Hebei in the Yellow and Huai River Valleys (Zone II) where almost one-half of the total Chinese wheat crop is produced.

This geographical differentiation for FHB resistance agrees with the prevalence of FHB. The Middle and Lower Yangtze River Valleys are the regularly hard-hit areas of FHB in China (Ma et al., 2020). The high disease pressure provides an appropriate environment for natural and artificial selection. Many FHB resistant or moderately resistant (MR) landraces were selected by local farmers, including Wangshuibai (in Jiangsu) and Chongyanghongmai (in Hubei) (Lu et al., 2001). The threat of FHB in this region also makes FHB resistance to be a priority of breeding objectives. Long-term breeding effort leads to the favorable alleles pyramiding and FHB resistance improvement. The rich resistant resources in this region, in turn, facilitate FHB resistance breeding. Indeed, Hubei and Jiangsu are the provinces where wheat clutivars showed the best FHB resistance according to the phenotypic results in the present study. FHB resistant germplasm from this region, such as Sumai 3 and Ning 7840 from Jiangsu, and Wuhan 1 from Hubei, was wildly used in global wheat breeding programs. For example, Sumai 3 is a major FHB resistant source for more than 20 modern wheat cultivars in the United States and Canada (Zhu et al., 2019; Hao et al., 2020). On the other hand, the high level of host resistance might be a driving force for the evolution of the pathogen. FHB resistant modern cultivars or elite lines identified from this region are valuable genetic resources in breeding for FHB resistance. Interestingly, many of these resistant cultivars could trace their resistance to Italian sources such as Mentana and Funo (Zhu et al., 2019), which might be due to different choices of breeders. To make good use of the resistance, molecular dissection of underlying QTL is urgent to be carried out.

Fusarium head blight was previously not a serious problem in the Yellow and Huai River Valleys but has increasingly become an important disease in recent years (Zhu et al., 2018). To improve FHB resistance levels, breeders here have started to use resistance sources from Jiangsu and Hubei, and local sources from Shaanxi, such as Xinong 2000, Xiaoyan 6, and Zhengmai 9023 (Zhu et al., 2019). Cultivars from Shaanxi generally have better FHB resistance than those from other provinces in the same agro-ecological zone, probably due to use of Sumai 3 and *Th. ponticum* as sources of FHB resistance in breeding (Xu et al., 1994; Zhu et al., 2019). However, the lack of consistent FHB development in breeding nurseries makes phenotypic selection difficult. More intensive selection approaches, such as dedicated FHB nurseries or molecular markers are essential for progress.

### Possible Mechanisms of Detected QTL for FHB Resistance

The interaction between host and pathogen of FHB is complex. To date, only *Fhb1* has been isolated, but its mechanism in FHB resistance remains unknown. Omics-based studies, along with targeted transgenic studies have shed insights into mechanisms involved in FHB resistance (Walter et al., 2010; Ma et al., 2020). Based on this knowledge, putative candidate genes were identified in the target QTL region. Genes encoding 12-oxophytodienoate reductase-like protein identified in the region of *QFhb.hbaas-1AS* may be involved in the biosynthesis or metabolism of signaling molecules oxylipin. Plant oxylipins such as JA could reduce *F. graminearum* growth and FHB symptoms (Qi et al., 2016). Genes encoding RLK, identified in the *QFhb.hbaas-1AS* and *QFhb.hbaas-5AL* regions, have been reported as DON and *Fusarium*-responsive and conferred type II resistance (Thapa et al., 2018). UGT could detoxify both DON and NIV produced by *F. graminearum* and enhance resistance to FHB in wheat and barley (Poppenberger et al., 2003; Li et al., 2017). The pathogenesis-related protein 1 (PR1) gene, identified in the *QFhb.hbaas-5AS* region, could increase type II resistance to FHB in wheat (Makandar et al., 2006). Glucan endo-1,3-beta-glucosidase is another PR protein PR2. Overexpression of *PR2* gene in wheat led to an enhanced resistance to FHB (Mackintosh et al., 2007). Defensin and other cysteine-rich proteins in cereal target the fungal membrane through interactions with phospholipids and sphingolipids (Walter et al., 2010), responding to pathogen invasion. To further study the functions of these candidate genes in FHB resistance, resistant lines in the WAPS population could be selected to examine the expression of those identified genes.

### Potential Use of *QFhb.hbaas-2DL*

The 2DL QTL was first mapped in the FHB moderately resistant line Wuhan 1 (Somers et al., 2003). Wangshuibai (Lin et al., 2006; Mardi et al., 2005) and CJ9306 (Jiang et al., 2007a, b) from Jiangsu, Sumai 3 derivatives DH 181 (Yang et al., 2005), SYN1, SHA3/CBRD (Lu et al., 2013) and Soru#1 (He et al., 2016) from CIMMYT, Swiss winter cultivar Arina (Paillard et al., 2004), and United States winter wheat line VA00W-38 (Liu et al., 2012) probably carry this QTL. Apparently, it has a relatively high frequency in the WAPS population. This QTL could be used as an important resistance source in combination with *Fhb1* and other detected loci. SSR marker *Xgwm539* can be used in MAS, but diagnostic PCR markers based on candidate genes *TaWRKY70*, *TaACT*, *TaDGK* and *TaGLI* (Kage et al., 2017a, b) would be more convenient for breeders.

### *QFhb.hbaas-1AS* Is Independent of Flowering Time and Plant Height

*QFhs.nau-1AS* linked to SSR markers *Xwmc24* and *Xbarc148* was identified in Chinese wheat line CJ9306 (Jiang et al., 2007a, b). The physical positions of these markers (27.3 and 52.2 Mb) are similar to the significant marker *IWB75039* (10.0 Mb) for *QFhb.hbaas-1AS* identified in this study. Both QTL probably represent the same resistance locus. Minor QTL on 1AS were also reported in susceptible cultivars Wheaton (Yu et al., 2008), Pirat (Holzapfel et al., 2008), and Pelikan (Häberle et al., 2010) but their relationships with *QFhb.hbaas-1AS* are unknown. *QFhb.hbaas-1AS* significantly reduced the FHB index in all years except 2015. This QTL was independent of flowering time and plant height, making it more flexible for use in breeding.

### *QFhb.hbaas-5AS*, *QFhb.hbaas-5AL*, and *QFhb.hbaas-7DS* Might Be New Loci for FHB Resistance

A QTL on 5A designated *Fhb5* was found in Wangshuibai, W14, and DH181 (Buerstmayr et al., 2009; Xue et al., 2011). The gene was flanked by markers *Xgwm304* and *Xgwm415* that span a physical interval between the physical positions of 105.4 Mb and 214.2 Mb, which is very close to the centromere of chromosome 5A (Ma et al., 2020). The physical position of the significant marker for *QFhb.hbaas-5AS* is at 9.6 Mb, indicating the difference of *QFhb.hbaas-5AS* from *Fhb5*. Recently, Steiner et al. (2019) finely mapped *Qfhs.ifa-5A* and separated it into two closely linked QTL *Qfhs.ifa-5Ac* (245.9–290.0 Mb) and *Qfhs.ifa-5AS* (70.7–119.9 Mb). Both distinguish from *QFhb.hbaas-5AS* mapped in the present study based on their physical positions.

Genes *Vrn-A1* and *Q* on chromosome 5AL were associated with FHB response (He et al., 2016). These genes are located at 587.4 and 650.1 Mb, respectively, whereas *QFhb.hbaas-5AL* is at 540.6 Mb, about 110 and 46.8 Mb away from them. Three QTL were detected on chromosome 5A from wheat cultivars Pirat, Apache (Holzapfel et al., 2008), and Arina (Paillard et al., 2004), but mapped to the physical positions (659.1, 659.1, and 698.2 Mb) different from *QFhb.hbaas-5AL*. Thus, *QFhb.hbaas-5AL* is probably a new locus for FHB resistance.

Very few QTL for FHB resistance have been identified on chromosome 7D. Cativelli et al. (2013) mapped a major QTL linked to SSR marker *Xcfd14* on 7DS in CIMMYT wheat cultivar Catbird, explaining 20% of the phenotypic variance. The physical position of *Xcfd14* is 263.0 Mb, whereas *QFhb.hbaas-7DS* is located around 23.5 Mb. Steed et al. (2016) reported that the resistance gene *Lr34*/*Yr18*/*Pm38* for multiple fungal pathogen might confer resistance to FHB. This gene is physically located at 47.4 Mb. It seems that *QFhb.hbaas-7DS* is different from the reported QTL/genes on this chromosome.

Validation of the putatively new QTL identified here will require analysis of bi-parental cross populations derived from representative genotypes in the WAPS population.

### Status and Perspective of *Fhb1* Exploitation in China

Sumai 3 was identified as the most FHB resistant cultivar in the present study. It has been used as a resistant parent in many crosses in China, but very few cultivars carry *Fhb1* (Zhu et al., 2019), which is probably due to the linkage drag associated with *Fhb1* in this donor. Apart from Sumai 3 and Ning 7840, only four cultivars, Ningmai 9 and its derivatives, in the present panel carry *Fhb1*. The four *Fhb1* carriers have much better yield and quality than Sumai 3. Due to Sumai 3′s poor agronomic performance, we recommend using Ningmai 9 derivatives as alternative donors of *Fhb1*. A back-cross strategy with MAS in large segregating populations will be crucial for the successful introduction of this gene in the Yellow and Huai River Valleys (Zone II). For a long term, other important genes/QTL can be gradually introduced into new cultivars with *Fhb1* through back-cross and MAS. Investigations of FHB resistance in indigenous accessions could also lead to the discovery of more sources of FHB resistance.

## Data Availability Statement

The datasets generated for this study can be found in EVA accession PRJEB36125.

## Author Contributions

ZZ performed the experiment and wrote the manuscript. LC and WZha contributed to genotyping. LY, WZhu, JuL, YL, and HT participated in the field trials. LF, JiL, and AR participated in marker development and the data analysis. CG, YH, XX, and ZH designed the experiment and assisted in writing the manuscript. All authors have reviewed the manuscript.

## Conflict of Interest

The authors declare that the research was conducted in the absence of any commercial or financial relationships that could be construed as a potential conflict of interest.

## Footnotes

1 http://www.wheatgenome.org

2 https://CRAN.R-project.org/package=CMplot

3 http://software.genetics.ucla.edu/admixture

4 http://zzlab.net/GAPIT

5 http://www.maizegenetics.net/tassel

6 https://wheat-urgi.versailles.inra.fr/Seq-Repository/BLAST

7 http://polymarker.tgac.ac.uk

8 http://plants.ensembl.org

## Abbreviations

15-AcDON: 15-O-acetyl-DON
3-AcDON: 3-O-acetyl-DON
AE: anther extrusion
ANOVA: analysis of variance
BLUE: best linear unbiased estimation
CAPS: cleaved amplified polymorphic sequences
CIMMYT: International Maize and Wheat Improvement Center
DON: deoxynivalenol
DON-3-G: deoxynivalenol-3-glucoside
FHB: Fusarium head blight
GWAS: genome-wide association study
*His* or *TaHRC*: histidine-rich calcium-binding
JA: jasmonic acid
KASP: kompetitive allele specific PCR
MAF: minor allele frequency
MAS: marker-assisted selection
MTA: marker-trait association
NIV: nivalenol
*PFT*: pore-forming toxin-like
PVE: phenotypic variation explained
QTL: quantitative trait loci
RLK: receptor-like kinase
SNP: single nucleotide polymorphism
UGT: UDP-glycosyltransferase
WAK: wall-associated receptor-like kinase
WAPS: Wheat Association Panel for Scab Research.
