# Supplementary material for: Genome-Wide Association Analysis of Fusarium Head Blight Resistance in Chinese Elite Wheat Lines
Source: Front Plant Sci. 2020 Feb 27;11:206. doi: 10.3389/fpls.2020.00206 (PMC7056811; doi:10.3389/fpls.2020.00206)
Supplement: Supplementary file 1 [file Data_Sheet_1.docx]

Table S1 Origin, putative QTL and BLUE values of FHB indices of wheat cultivars and elite lines in the WAPS population

| No. | Cultivar | Wheat zone ^a^ | Origin | *Qfhb.hbaas-1AS*^b^ | *Qfhb.hbaas-2DL*^b^ | *Qfhb.hbaas-5AS*^b^ | *Qfhb.hbaas-5AL*^b^ | *Qfhb.hbaas-7DS*^b^ | BLUE of FHB indexes (%) |
| --- | --- | --- | --- | --- | --- | --- | --- | --- | --- |
| 1 | Beijing 0045 | I | Beijing | - | + | + | + | - | 43.8 |
| 2 | Jingdong 8 | I | Beijing | - | - | + | - | - | 39.8 |
| 3 | Jingdong 17 | I | Beijing | - | - | + | + | - | 56.4 |
| 4 | Lunxuan 987 | I | Beijing | - | - | + | + | - | 35.9 |
| 5 | Zhongmai 9 | I | Beijing | - | - | + | + | - | 50.3 |
| 6 | Zhongmai 12 | I | Beijing | - | - | + | + | - | 57.5 |
| 7 | Zhongnong 2 | I | Beijing | - | - | + | - | - | 35.3 |
| 8 | Wanmai 38 | II | Anhui | - | - | + | + | - | 33.7 |
| 9 | Wanmai 50 | II | Anhui | - | - | + | + | - | 40.4 |
| 10 | Wanmai 52 | II | Anhui | - | - | + | + | - | 44.5 |
| 11 | Han 3475 | II | Hebei | - | - | - | - | - | 70.4 |
| 12 | Han 5316 | II | Hebei | - | - | + | - | - | 69.3 |
| 13 | Han 6172 | II | Hebei | - | - | + | + | - | 61.5 |
| 14 | Heng 115 | II | Hebei | + | + | + | - | - | 56.9 |
| 15 | Heng 136 | II | Hebei | - | - | + | - | - | 61.4 |
| 16 | Heng 4422 | II | Hebei | - | - | + | + | - | 57.2 |
| 17 | Hengguan 35 | II | Hebei | - | - | - | - | - | 80.1 |
| 18 | Jimai 30 | II | Hebei | - | - | + | + | - | 34.4 |
| 19 | Jimai 38 | II | Hebei | - | - | - | + | - | 59.3 |
| 20 | Kenong 199 | II | Hebei | - | + | - | - | - | 71.2 |
| 21 | Kenong 9204 | II | Hebei | - | - | - | - | - | 56.2 |
| 22 | Shijiazhuang 8 | II | Hebei | - | - | - | - | - | 72.1 |
| 23 | 04 zhong 36 | II | Henan | - | - | - | - | - | 54.9 |
| 24 | Aikang 58 | II | Henan | - | - | - | - | - | 65.6 |
| 25 | Aizao 781-99 selection | II | Henan | - | - | + | + | - | 53.0 |
| 26 | Bainong 160 | II | Henan | - | - | + | - | - | 71.1 |
| 27 | Fanmai 5 | II | Henan | - | - | - | + | - | 62.7 |
| 28 | Jinfeng 3 | II | Henan | - | - | + | - | - | 60.0 |
| 29 | Kaimai 18 | II | Henan | - | U | + | - | - | 59.9 |
| 30 | Keda 9612 | II | Henan | - | - | - | - | - | 74.8 |
| 31 | Luo 4-168 | II | Henan | - | - | + | - | - | 70.4 |
| 32 | Luohan 2 | II | Henan | - | - | + | - | - | 58.8 |
| 33 | Luohan 6 | II | Henan | - | + | + | + | - | 56.7 |
| 34 | Luohan 7 | II | Henan | - | - | + | + | - | 59.5 |
| 35 | Luomai 21 | II | Henan | - | - | + | - | - | 56.4 |
| 36 | Luomai 6010 | II | Henan | + | - | + | + | - | 33.5 |
| 37 | Luoxin 998 | II | Henan | - | - | - | - | - | 74.8 |
| 38 | Pingan 3 | II | Henan | - | - | + | - | - | 59.6 |
| 39 | Pingan 6 | II | Henan | - | - | - | + | - | 54.8 |
| 40 | Pumai 10 | II | Henan | - | - | - | - | - | 61.1 |
| 41 | Wenmai 6 | II | Henan | - | - | + | - | - | 65.3 |
| 42 | Wenmai 7 | II | Henan | - | - | + | - | - | 74.2 |
| 43 | Wenmai 18 | II | Henan | - | - | + | - | - | 63.3 |
| 44 | Wenmai 19 | II | Henan | - | - | + | - | - | 68.2 |
| 45 | Xiangmai 99 | II | Henan | - | - | + | - | - | 67.7 |
| 46 | Xinmai 11 | II | Henan | - | - | - | - | - | 43.2 |
| 47 | Xinmai 13 | II | Henan | - | - | - | - | - | 67.6 |
| 48 | Xinmai 16 | II | Henan | - | - | - | - | - | 60.6 |
| 49 | Xinmai 18 | II | Henan | - | - | - | - | - | 54.5 |
| 50 | Xinmai 19 | II | Henan | - | - | + | - | - | 54.6 |
| 51 | Xinmai 20 | II | Henan | - | + | + | + | - | 37.7 |
| 52 | Xinmai 22 | II | Henan | - | - | + | - | - | 34.8 |
| 53 | Xinmai 26 | II | Henan | - | - | - | - | - | 50.6 |
| 54 | Xinmai 208 | II | Henan | - | - | + | + | - | 60.4 |
| 55 | Xinmai 9817 | II | Henan | - | - | + | - | - | 42.6 |
| 56 | Xinmai 9817 selection | II | Henan | - | - | + | + | - | 37.7 |
| 57 | Xuke 1 | II | Henan | - | - | - | - | - | 64.4 |
| 58 | Yanzhan 4110 | II | Henan | - | - | + | + | - | 46.8 |
| 59 | Yumai 10 | II | Henan | - | - | + | + | + | 57.8 |
| 60 | Yumai 38 | II | Henan | - | - | + | - | - | 49.2 |
| 61 | Yumai 48 | II | Henan | - | - | - | - | - | 46.0 |
| 62 | Yumai 49-168 | II | Henan | - | - | + | - | - | 74.2 |
| 63 | Yumai 49-198 | II | Henan | - | - | + | - | - | 65.5 |
| 64 | Yumai 52 | II | Henan | - | - | + | - | - | 70.1 |
| 65 | Yumai 69 | II | Henan | - | - | - | - | - | 55.8 |
| 66 | Yumai 70 | II | Henan | - | - | + | - | - | 40.7 |
| 67 | Yumai 70-36 | II | Henan | - | - | + | - | - | 40.3 |
| 68 | Yunong 035 | II | Henan | - | - | + | - | - | 47.8 |
| 69 | Yunong 202 | II | Henan | - | - | - | + | - | 38.4 |
| 70 | Zhengmai 004 | II | Henan | - | + | + | - | - | 54.1 |
| 71 | Zhengmai 98 | II | Henan | - | + | - | - | - | 51.9 |
| 72 | Zhengmai 366 | II | Henan | - | - | + | + | - | 68.3 |
| 73 | Zhengmai 9023 | II | Henan | - | - | + | - | + | 29.8 |
| 74 | Zhengmai 9694 | II | Henan | - | - | + | - | - | 60.3 |
| 75 | Zhengnong 17 | II | Henan | - | - | - | + | - | 80.3 |
| 76 | Zhengyumai 958 | II | Henan | - | - | + | - | + | 44.9 |
| 77 | Zhengyumai 9987 | II | Henan | - | - | - | - | - | 56.4 |
| 78 | Zhongmai 1 | II | Henan | - | - | + | - | - | 62.8 |
| 79 | Zhongyu 10 | II | Henan | - | - | + | - | - | 58.3 |
| 80 | Zhoumai 16 | II | Henan | - | - | - | + | - | 64.2 |
| 81 | Zhoumai 17 | II | Henan | - | - | + | - | - | 73.8 |
| 82 | Zhoumai 18 | II | Henan | - | - | - | - | - | 54.8 |
| 83 | Zhoumai 22 | II | Henan | - | - | - | + | - | 68.3 |
| 84 | Zhoumai 23 | II | Henan | - | - | - | - | - | 56.6 |
| 85 | Huaimai 17 | II | Jiangsu | - | - | + | + | + | 31.9 |
| 86 | Huaimai 18 | II | Jiangsu | - | - | - | + | - | 58.7 |
| 87 | Huaimai 20 | II | Jiangsu | - | + | - | + | - | 32.2 |
| 88 | Lianmai 1 | II | Jiangsu | - | - | - | + | - | 45.6 |
| 89 | Lianmai 2 | II | Jiangsu | - | - | - | + | - | 41.8 |
| 90 | Xumai 27 | II | Jiangsu | - | - | + | - | + | 58.2 |
| 91 | Xumai 29 | II | Jiangsu | - | - | + | - | - | 66.4 |
| 92 | Xumai 216 | II | Jiangsu | - | - | + | - | - | 51.6 |
| 93 | Liken 2 | II | Shaanxi | - | - | + | + | + | 37.3 |
| 94 | Qinnong 142 | II | Shaanxi | - | - | - | U | U | 52.1 |
| 95 | Shaan 253 | II | Shaanxi | - | - | + | - | + | 65.8 |
| 96 | Shaan 627 | II | Shaanxi | - | - | - | - | - | 47.6 |
| 97 | Shaan 715 | II | Shaanxi | - | - | - | - | - | 43.2 |
| 98 | Shaanmai 139 | II | Shaanxi | - | - | - | - | - | 61.7 |
| 99 | Shaanmai 150 | II | Shaanxi | - | - | U | - | U | 39.3 |
| 100 | Shaanmai 159 | II | Shaanxi | - | - | - | - | - | 45.9 |
| 101 | Shaannong 78 | II | Shaanxi | - | - | - | - | + | 43.5 |
| 102 | Shaannong 138 | II | Shaanxi | - | - | - | - | - | 42.6 |
| 103 | Shaannong 757 | II | Shaanxi | - | - | + | - | - | 38.8 |
| 104 | Xiaoyan 6 | II | Shaanxi | - | - | U | U | U | 33.2 |
| 105 | Xiaoyan 22 | II | Shaanxi | - | - | + | - | + | 60.7 |
| 106 | Xiaoyan 107 | II | Shaanxi | - | U | + | + | - | 41.4 |
| 107 | Xiaoyan 166 | II | Shaanxi | - | - | - | - | + | 41.7 |
| 108 | Xinong 88 | II | Shaanxi | - | - | + | + | - | 55.8 |
| 109 | Xinong 979 | II | Shaanxi | - | - | + | + | - | 56.5 |
| 110 | Xinong 2000 | II | Shaanxi | - | - | - | - | + | 28.1 |
| 111 | Xinong 3517 | II | Shaanxi | - | - | + | + | - | 38.0 |
| 112 | Xinong 9871 | II | Shaanxi | - | - | - | - | - | 68.6 |
| 113 | Yuanfeng 175 | II | Shaanxi | - | - | - | - | - | 59.7 |
| 114 | Hemai 13 | II | Shandong | - | - | + | + | - | 58.3 |
| 115 | Jimai 19 | II | Shandong | - | - | + | + | - | 50.5 |
| 116 | Jimai 20 | II | Shandong | - | - | - | + | - | 54.7 |
| 117 | Jimai 21 | II | Shandong | - | - | - | - | - | 52.9 |
| 118 | Jimai 22 | II | Shandong | - | - | - | + | - | 47.2 |
| 119 | Jinan 17 | II | Shandong | - | - | - | + | - | 41.0 |
| 120 | Jinan 17 selection | II | Shandong | - | - | + | + | - | 46.9 |
| 121 | Jining 16 | II | Shandong | - | - | + | - | - | 57.0 |
| 122 | Liangxing 99 | II | Shandong | - | - | - | + | - | 57.2 |
| 123 | Lumai 21 | II | Shandong | - | - | + | + | - | 56.4 |
| 124 | Lunong 116 | II | Shandong | - | - | - | - | - | 48.3 |
| 125 | Qingfeng 1 | II | Shandong | - | - | - | + | - | 59.0 |
| 126 | Shannong 15 | II | Shandong | - | - | - | + | - | 45.2 |
| 127 | Shannong 16 | II | Shandong | + | - | - | + | - | 44.8 |
| 128 | Shannong 189 | II | Shandong | + | - | + | + | - | 56.5 |
| 129 | Shannong 664 | II | Shandong | - | - | - | + | - | 58.2 |
| 130 | Shannong 8355 | II | Shandong | - | - | - | - | - | 88.6 |
| 131 | Tainong 18 | II | Shandong | - | - | - | - | - | 57.8 |
| 132 | Taishan 21 | II | Shandong | - | - | + | + | - | 48.2 |
| 133 | Taishan 23 | II | Shandong | - | - | + | + | - | 63.4 |
| 134 | Weimai 8 | II | Shandong | - | - | - | + | - | 44.2 |
| 135 | Yan 2415 | II | Shandong | - | - | - | + | - | 35.9 |
| 136 | Yan 5158 | II | Shandong | - | - | - | + | - | 64.2 |
| 137 | Yan 5286 | II | Shandong | - | - | + | U | - | 37.8 |
| 138 | Yannong 19 | II | Shandong | - | + | - | - | - | 47.3 |
| 139 | Yannong 21 | II | Shandong | - | - | + | + | - | 44.7 |
| 140 | Yannong 22 | II | Shandong | - | - | + | + | + | 53.4 |
| 141 | Yannong 24 | II | Shandong | - | - | - | + | - | 54.9 |
| 142 | Chang 6359 | II | Shanxi | - | - | - | - | - | 41.8 |
| 143 | Lin Y867 | II | Shanxi | - | - | + | + | - | 39.8 |
| 144 | Lin Y7287 | II | Shanxi | - | - | + | + | - | 60.2 |
| 145 | Linfen 137 | II | Shanxi | - | - | - | + | - | 60.3 |
| 146 | Linfen 138 | II | Shanxi | - | - | - | - | - | 46.0 |
| 147 | Linyou 2069 | II | Shanxi | - | - | - | + | - | 54.5 |
| 148 | E 07901 | III | Hubei | - | - | - | + | + | 31.4 |
| 149 | Een 1 | III | Hubei | - | - | + | + | - | 28.8 |
| 150 | Een 5 | III | Hubei | - | - | - | - | - | 37.4 |
| 151 | Een 6 | III | Hubei | - | - | + | - | - | 35.5 |
| 152 | Emai 11 | III | Hubei | + | + | + | + | - | 37.0 |
| 153 | Emai 12 | III | Hubei | - | - | + | - | - | 35.7 |
| 154 | Emai 18 | III | Hubei | + | + | + | + | - | 28.9 |
| 155 | Emai 23 | III | Hubei | - | - | + | - | - | 28.8 |
| 156 | Emai 27 | III | Hubei | - | + | + | - | - | 30.4 |
| 157 | Emai 352 | III | Hubei | - | - | + | - | - | 18.6 |
| 158 | Emai 580 | III | Hubei | + | + | + | + | - | 29.6 |
| 159 | Emai 596 | III | Hubei | - | - | + | - | + | 23.9 |
| 160 | Hua 2533 | III | Hubei | - | - | + | - | - | 66.4 |
| 161 | Hua 2566 | III | Hubei | - | + | + | - | - | 17.2 |
| 162 | Jingfumai 1 | III | Hubei | - | + | + | - | - | 20.6 |
| 163 | Jingmai 103 | III | Hubei | - | - | - | - | - | 37.9 |
| 164 | Jingzhou 66 | III | Hubei | - | + | + | + | - | 15.1 |
| 165 | Wuhan 1 | III | Hubei | - | + | + | - | - | 27.4 |
| 166 | Xiangmai 25 | III | Hubei | + | + | + | + | - | 9.7 |
| 167 | Xiangmai 55 | III | Hubei | + | + | + | + | - | 11.3 |
| 168 | CJ9306 | III | Jiangsu | + | + | + | - | - | 21.3 |
| 169 | Ning 7840 | III | Jiangsu | - | - | + | + | - | 13.4 |
| 170 | Ningmai 8 | III | Jiangsu | - | + | + | - | - | 39.2 |
| 171 | Ningmai 9 | III | Jiangsu | - | - | + | + | - | 29.4 |
| 172 | Ningmai 11 | III | Jiangsu | - | + | - | - | - | 31.4 |
| 173 | Ningmai 13 | III | Jiangsu | - | - | U | + | - | 42.8 |
| 174 | Ningmai 16 | III | Jiangsu | - | + | + | - | - | 37.1 |
| 175 | Sumai 3 | III | Jiangsu | - | + | + | + | - | 5.3 |
| 176 | Yang 05-117 | III | Jiangsu | - | + | + | - | - | 21.2 |
| 177 | Yang 06-144 | III | Jiangsu | + | + | + | - | - | 20.2 |
| 178 | Yang 06G86 | III | Jiangsu | + | + | + | - | - | 11.0 |
| 179 | Yang 07-129 | III | Jiangsu | U | + | - | + | - | 20.8 |
| 180 | Yang 07-15 | III | Jiangsu | - | + | - | - | - | 42.8 |
| 181 | Yang 07-44 | III | Jiangsu | - | + | - | - | + | 19.5 |
| 182 | Yang 07-49 | III | Jiangsu | - | + | + | - | + | 23.9 |
| 183 | Yang 07-81 | III | Jiangsu | + | - | + | - | U | 11.6 |
| 184 | Yangfumai 2 | III | Jiangsu | - | + | + | - | - | 23.9 |
| 185 | Yangmai 11 | III | Jiangsu | - | + | + | - | - | 17.9 |
| 186 | Yangmai 12 | III | Jiangsu | - | + | + | - | - | 14.6 |
| 187 | Yangmai 13 | III | Jiangsu | - | + | - | - | - | 31.1 |
| 188 | Yangmai 14 | III | Jiangsu | - | + | + | + | - | 12.0 |
| 189 | Yangmai 15 | III | Jiangsu | + | + | - | - | - | 44.1 |
| 190 | Yangmai 16 | III | Jiangsu | + | + | + | + | - | 15.1 |
| 191 | Yangmai 17 | III | Jiangsu | + | + | + | - | - | 17.7 |
| 192 | Yangmai 20 | III | Jiangsu | - | + | - | - | - | 25.7 |
| 193 | Yangmai 22 | III | Jiangsu | - | + | - | - | - | 22.3 |
| 194 | Yangmai 158 | III | Jiangsu | - | + | + | - | - | 16.4 |
| 195 | Zhenmai 5 | III | Jiangsu | - | + | + | - | - | 21.6 |
| 196 | Zhenmai 6 | III | Jiangsu | - | + | + | - | - | 16.3 |
| 197 | Zhenmai 168 | III | Jiangsu | - | - | + | - | - | 29.5 |
| 198 | Chuanmai 42 | IV | Sichuan | - | - | - | + | - | 46.2 |
| 199 | Chuanmai 42 selection | IV | Sichuan | - | - | - | + | - | 53.0 |
| 200 | Chuanmai 43 | IV | Sichuan | - | - | - | + | - | 50.6 |
| 201 | Chuanmai 50 | IV | Sichuan | - | - | - | - | - | 50.6 |
| 202 | Chuanmai 51 | IV | Sichuan | - | - | - | + | - | 31.8 |
| 203 | Chuanmai 52 | IV | Sichuan | - | - | + | - | - | 50.8 |
| 204 | Mianmai 37 | IV | Sichuan | - | - | - | - | - | 53.1 |
| 205 | Mianmai 42 | IV | Sichuan | + | - | - | - | - | 34.1 |
| 206 | Mianyang 99-3 | IV | Sichuan | - | - | + | - | - | 51.2 |
| 207 | Mianyang 99-7 | IV | Sichuan | - | - | + | - | - | 20.2 |
| 208 | Mianmai 185 | IV | Sichuan | - | - | - | - | - | 54.8 |
| 209 | Mianmai 1403 | IV | Sichuan | - | + | - | - | - | 69.2 |
| 210 | Neimai 8 | IV | Sichuan | - | - | - | - | - | 47.2 |
| 211 | Shuangkang 7438 | IV | Sichuan | - | - | - | - | - | 64.4 |
| 212 | Xikemai 2 | IV | Sichuan | - | - | - | - | - | 59.7 |
| 213 | Xikemai 4 | IV | Sichuan | - | - | - | - | - | 57.6 |
| 214 | Xikemai 5 | IV | Sichuan | - | - | - | + | - | 54.1 |
| 215 | XK0106-1-0806 | IV | Sichuan | - | + | - | - | - | 39.7 |
| 216 | Lantian 12 | VIII | Gansu | + | - | + | + | + | 36.2 |
| 217 | Lantian 13 | VIII | Gansu | - | - | + | - | - | 26.5 |
| 218 | Lantian 15 | VIII | Gansu | - | - | - | - | - | 62.0 |
| 219 | Lantian 17 | VIII | Gansu | - | + | - | - | - | 34.9 |
| 220 | Lantian 18 | VIII | Gansu | + | - | + | - | - | 24.9 |
| 221 | Lantian 21 | VIII | Gansu | + | U | + | - | - | 51.1 |
| 222 | Lantian 22 | VIII | Gansu | - | - | + | + | - | 21.5 |
| 223 | Lantian 23 | VIII | Gansu | - | - | + | - | - | 53.3 |
| 224 | Lantian 26 | VIII | Gansu | - | - | + | - | - | 46.9 |
| 225 | Ningchun 4 | VIII | Ningxia | + | - | + | + | - | 74.3 |
| 226 | Ningchun 43 | VIII | Ningxia | - | - | + | + | - | 75.9 |
| 227 | Ningchun 47 | VIII | Ningxia | - | - | + | - | - | 62.5 |
| 228 | Ningdong 10 | VIII | Ningxia | - | - | + | + | - | 52.5 |
| 229 | Ningdong 11 | VIII | Ningxia | - | - | + | + | - | 42.1 |
| 230 | Gamenya |  | Australia | U | - | + | U | U | 73.8 |
| 231 | CROC_1/AE.SQUARROSA (205)//KAUZ/3/SASIA/4/TROST |  | CIMMYT | + | - | + | + | - | 43.3 |
| 232 | MONARCA F2007/KRONSTAD F2004 |  | CIMMYT | - | - | + | + | - | 39.4 |
| 233 | PBW343*2/KUKUNA//PBW343*2/KUKUNA/3/PBW343 |  | CIMMYT | - | - | - | + | - | 40.9 |
| 234 | KS82W418/SPN//WBLL1/3/BERKUT |  | CIMMYT | - | - | - | + | - | 52.3 |
| 235 | CNDO/R143//ENTE/MEXI75/3/AE.SQ/4/2*FCT/5/KAUZ*2/YACO//KAUZ/6/BERKUT |  | CIMMYT | - | - | + | + | - | 38.7 |
| 236 | T.DICOCCON PI94625/AE.SQUARROSA (372)//TUI/CLMS/3/2*PASTOR/4/EXCALIBUR |  | CIMMYT | + | - | - | + | + | 32.5 |
| 237 | NG8675/CBRD//MILAN/3/SAUAL/6/CNDO/R143//ENTE/MEXI_2/3/AEGILOPS SQUARROSA (TAUS)/4/WEAVER/5/2*PASTOR |  | CIMMYT | U | + | + | U | - | 47.3 |
| 238 | Mayoor |  | CIMMYT | U | - | + | + | - | 27.5 |
| 239 | Ocoroni |  | CIMMYT | - | - | + | + | - | 45.7 |
| 240 | SYN1 |  | CIMMYT | U | - | + | + | - | 27.3 |

^a^ I: Northern Winter Wheat Zone; II: Yellow and Huai River Valleys Facultative Wheat Zone; III: Middle and Lower Yangtze Valleys Autumn-Sown Spring Wheat Zone; IV: Southwestern Autumn-Sown Spring Wheat Zone; V: Southern Autumn-sown Spring Wheat Zone; VI: Northeastern Spring Wheat Zone; VII: Northern Spring Wheat Zone; VIII: Northwestern Spring Wheat Zone; IX: Qinghai-Tibetan Plateau Spring-Winter Wheat Zone; X: Xinjiang Winter-Spring Wheat Zone.

^b^ “+”, resistance allele of the corresponding QTL; “-”, susceptibility allele of the corresponding QTL; “U”, uncertain.

Table S2 Correlation coefficients of Fusarium head blight indices of the WAPS (Wheat Association Panel for Scab Research) population among different years in Wuhan

| Year | 2014 | 2015 | 2016 |
| --- | --- | --- | --- |
| 2015 | 0.47** |  |  |
| 2016 | 0.66** | 0.41** |  |
| 2017 | 0.41** | 0.31** | 0.52** |

** Significant at *P* < 0.01

Table S3 Number of markers, physical distance coverage and marker density for the 21 wheat chromosomes and A, B and D genomes

| Chromosome | Number of markers | Coverage (Mb) | Marker density (Mb/marker) |
| --- | --- | --- | --- |
| 1A | 996 | 592.1 | 0.595 |
| 2A | 831 | 778.1 | 0.936 |
| 3A | 770 | 749.0 | 0.973 |
| 4A | 584 | 741.7 | 1.270 |
| 5A | 905 | 708.4 | 0.783 |
| 6A | 910 | 617.4 | 0.678 |
| 7A | 782 | 736.2 | 0.941 |
| 1B | 1,292 | 687.8 | 0.532 |
| 2B | 1,050 | 800.0 | 0.762 |
| 3B | 878 | 829.3 | 0.945 |
| 4B | 483 | 672.7 | 1.393 |
| 5B | 1098 | 712.8 | 0.649 |
| 6B | 959 | 720.8 | 0.752 |
| 7B | 828 | 750.5 | 0.906 |
| 1D | 644 | 495.0 | 0.769 |
| 2D | 359 | 650.9 | 1.813 |
| 3D | 257 | 613.9 | 2.389 |
| 4D | 106 | 508.6 | 4.798 |
| 5D | 287 | 562.1 | 1.959 |
| 6D | 300 | 471.5 | 1.572 |
| 7D | 259 | 637.2 | 2.460 |
| A genome | 5,778 | 4,922.9 | 0.852 |
| B genome | 6,588 | 5,174.0 | 0.785 |
| D genome | 2,212 | 3,939.2 | 1.781 |
| Total | 14,578 | 14,036.0 | 0.963 |

Table S4 Protocols for use of markers *FHB-1AS-PCR*, *FHB-5AS-KASP* and *FHB-5AL-CAPS*

| Marker | PCR system^a^ | PCR program | Detection | Result calling | |
| --- | --- | --- | --- | --- | --- |
|  |  |  |  | Resistance | Susceptibility |
| *FHB-1AS-PCR* | 20 μL: 10 μL 2 × PCR Mix, 2 μL DNA template (50 ng/μL), 0.5 μL each of the forward and reverse primers (10 μmol/L), and 7 μL ddH_2_O | 5 min at 95°C; 35 cycles (94°C for 30 s, 60°C for 30 s, 72°C for 1 min 30 s); final extension 5 min at 72°C | 1.5% agarose gel | no band | 389 bp band |
| *FHB-5AS-KASP* | Dry DNA method, 4 μL: 2 μL KASP Master Mix, 0.045 μL primer mix (A:B:C:ddH_2_O = 12:12:30:45), 2.0 μL DNA template (30 ng/μL) and 1.955 μL ddH_2_O | 15 min at 95°C; 10 touchdown cycles with a 0.6°C drop for annealing and elongation per cycle (95°C for 20 s, 65°C for 30 s); 32 cycles (95°C 20 s, 57°C 1 min) | PCR product were detected by PHERAstar *^Plus^* and then analyzed using KlusterCaller software. | red dot | blue dot |
| *FHB-5AL-CAPS* | 20 μL: 10 μL 2 × PCR Mix, 2 μL DNA template (50 ng/μL), 0.5 μL each of the forward and reverse primers (10 μmol/L), and 7 μL ddH_2_O | 5 min at 95°C; 35 cycles (94°C for 30 s, 60°C for 30 s, 72°C for 1 min 30 s); final extension 5 min at 72°C | PCR product was digested with *Age* I overnight at 37°C in a 10 μL system (5 μL PCR product, 1 μL 10 × buffer, 0.25 μL *Age* I, and 3.75 μL ddH2O) and then detected by 1.5% agarose gel. | 334 bp band | 213 bp and 121 bp bands |

^a^ 2 × PCR Mix: 0.1 U/μL *Taq* DNA polymerase, 500 μmol/L dNTPs, 20 mmol/L Tri-HCl, 10 mmol/L KCl, 3 mmol/L MgCl_2_.

Table S5 Candidate genes for Fusarium head blight resistance QTL *QFhb.hbaas-1AS*, *QFhb.hbaas-5AS*, *QFhb.hbaas-5AL*, and *QFhb.hbaas-7DS*

| QTL | Gene ID | Position (Mb) | Annotation^a^ |
| --- | --- | --- | --- |
| *QFhb.hbaas-1AS* | TraesCS1A01G015500 | 8.3 | 12-oxophytodienoate reductase-like protein |
|  | TraesCS1A01G015600 | 8.3 | 12-oxophytodienoate reductase-like protein |
|  | TraesCS1A01G015700 | 8.3 | 12-oxophytodienoate reductase-like protein |
|  | TraesCS1A01G015800 | 8.3 | 12-oxophytodienoate reductase-like protein |
|  | TraesCS1A01G017700 | 9.2 | Receptor-like protein kinase |
|  | TraesCS1A01G017900 | 9.4 | Receptor-like kinase |
|  | TraesCS1A01G018000 | 9.4 | Receptor-like kinase protein |
|  | TraesCS1A01G018500 | 9.5 | Receptor-like kinase |
|  | TraesCS1A01G018600 | 9.6 | Receptor-like kinase |
|  | TraesCS1A01G019500 | 9.8 | UDP-glycosyltransferase |
|  | TraesCS1A01G019600 | 9.8 | UDP-glycosyltransferase |
|  | TraesCS1A01G023000 | 11.4 | cysteine-rich receptor-like protein kinase 19 |
| *QFhb.hbaas-5AS* | TraesCS5A01G012900 | 8.7 | Pathogenesis-related protein 1 |
|  | TraesCS5A01G013500 | 9.3 | Pathogenesis-related protein 1 |
| *QFhb.hbaas-5AL* | TraesCS5A01G329600 | 538.6 | Glucan endo-1,3-beta-glucosidase |
|  | TraesCS5A01G329700 | 538.8 | Glucan endo-1,3-beta-glucosidase |
|  | TraesCS5A01G330100 | 539.5 | Defensin |
|  | TraesCS5A01G330500 | 540.1 | Receptor-like kinase |
| *QFhb.hbaas-7DS* | TraesCS7D01G045000 | 22.8 | Protein enhanced disease resistance 2-like |

^a^Gene annotations were referred to IWGSC RefSeq annotation v1.0 (www.wheatgenome.org).

Fig. S1 Frequency distribution of Fusarium head blight (FHB) indices of 240 wheat cultivars and lines in 2014–2017 and for the BLUE values across four years

Fig. S2 Sample numbers and BLUE values of Fusarium head blight indices at the province level for the WAPS population

BJ, Beijing; HE, Hebei; SD, Shandong; SX, Shanxi; SN, Shaanxi, NX, Ningxia; GS, Gansu; JS, Jiangsu; AH, Anhui; HA, Henan; HB, Hubei; SC, Sichuan. Numeral in each province represents the number of cultivars (lines) sampled. Different colors represent corresponding FHB indices according to the legend. Ten CIMMYT lines are not shown in this figure.


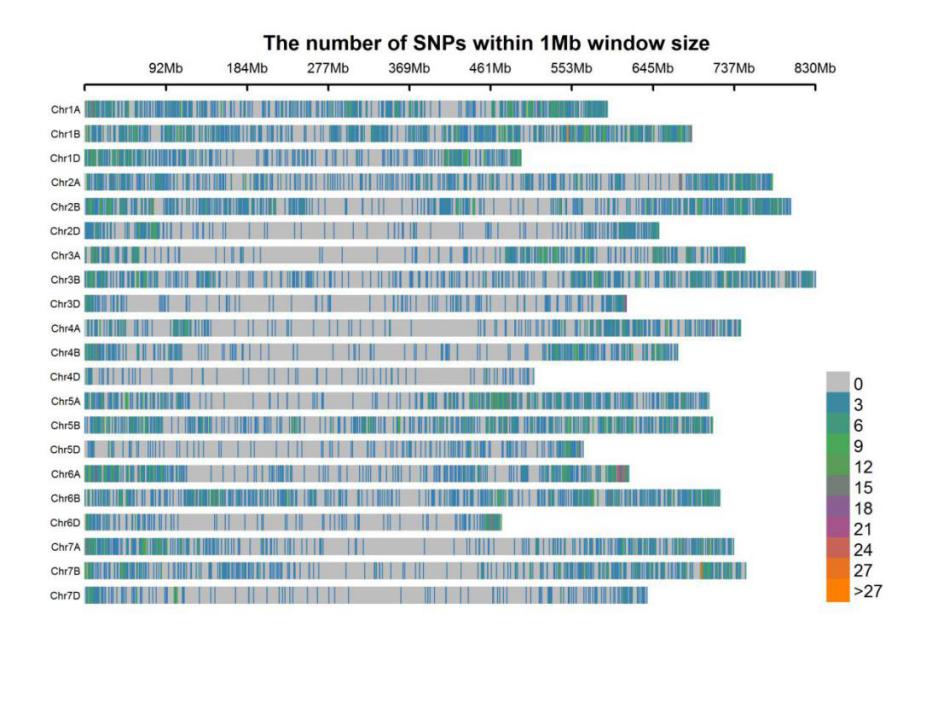


Fig. S3 Marker density of each chromosome of the WAPS population genotyped using the 90K SNP array. Different colors represent the corresponding number of SNP within 1 Mb distance according to the legend.

a


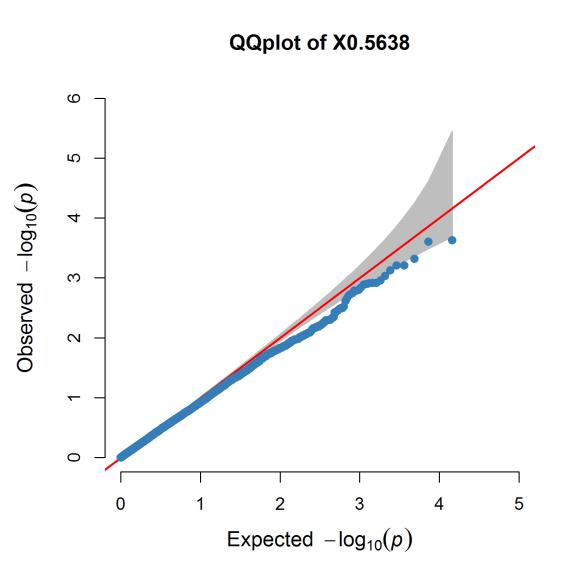

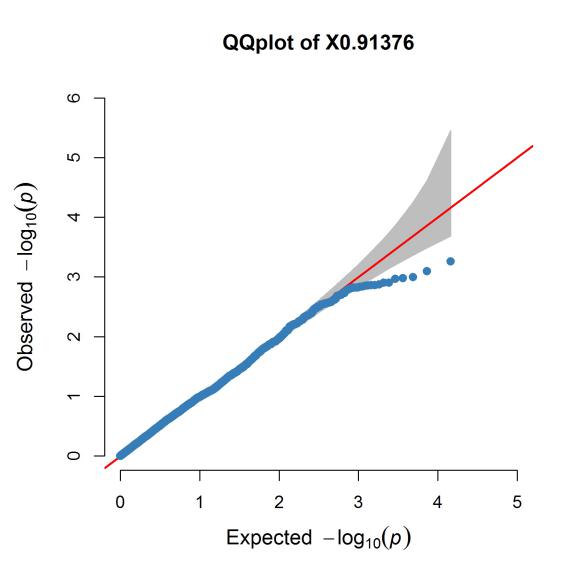


b

c

d


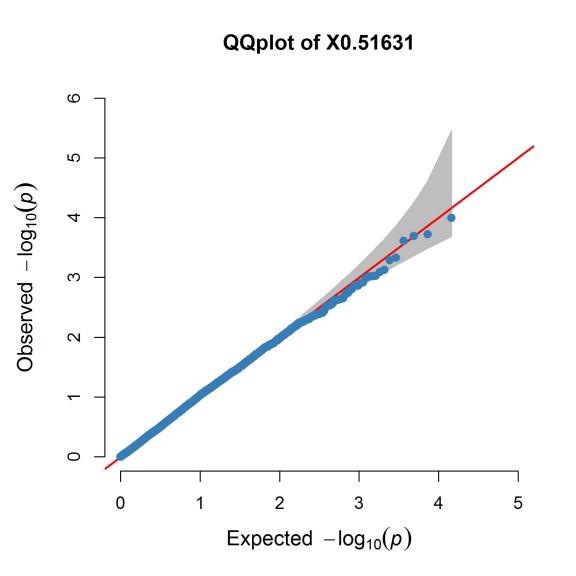

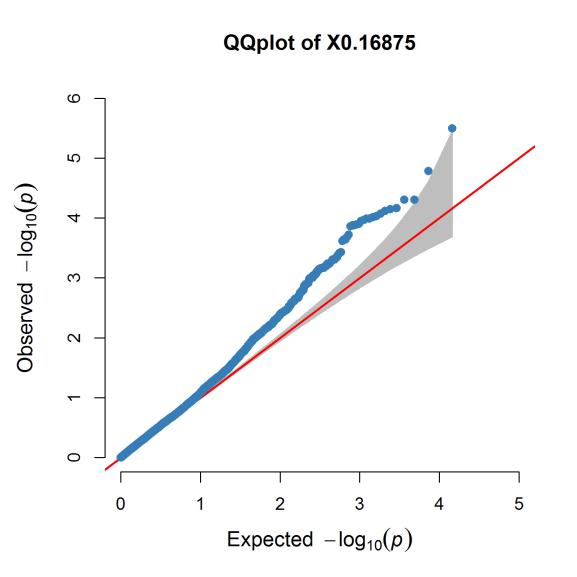


e


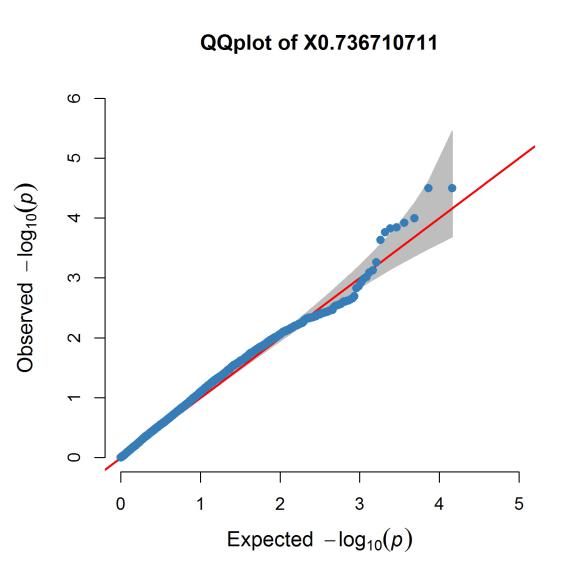


Fig. S4 Quantile-quantile (Q-Q) plots for FHB indices in 240 wheat accessions using the mixed linear model (MLM) in Tassel v5.0. (a) 2014; (b) 2015; (c) 2016; (d) 2017; (e) BLUE

Fig. S5 Allelic frequency and geographical distribution of resistance alleles of the five stable QTL

Numerals following QTL name represent the number of accessions carrying the respective resistance allele. Numerals inside pie chart sections represent number of accessions carrying the resistance allele with different origins. Alleles of wheat accessions for each QTL were determined by representative markers.


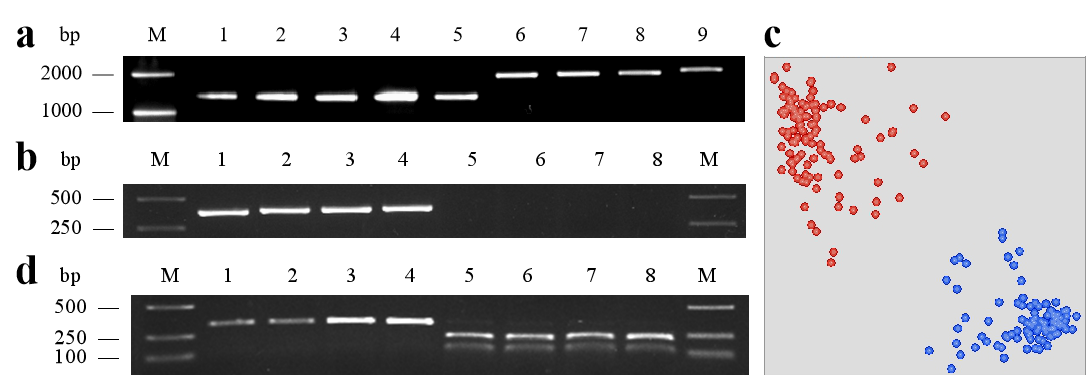


Fig. S6 Profiles of markers *His-InDel*, *FHB-1AS-PCR*, *FHB-5AS-KASP* and *FHB-5AL-CAPS for Fhb1*, *QFhb.hbaas-1AS*, *QFhb.hbaas-5AS* and *QFhb.hbaas-5AL*, respectively. (a) *His-InDel*, 1–5: lines with resistance allele *Fhb1*; 6–9: lines with susceptibility allele of *fhb1*; (b) *FHB-1AS-PCR*, 1–4: lines with susceptibility allele of *QFhb.hbaas-1AS*; 5–8: lines with resistance allele of *QFhb.hbaas-1AS*; (c) *FHB-5AS-KASP*, red and blue dots represented resistance and susceptibility alleles of *QFhb.hbaas-5AS*, respectively; (d) *FHB-5AL-CAPS*, 1–4: lines with resistance allele of *QFhb.hbaas-5AL*; 5–8: lines with susceptibility allele of *QFhb.hbaas-5A*
